# Evaluation and Integration of Genetic Signature for Prediction Risk of Nasopharyngeal Carcinoma in Southern China

**DOI:** 10.1155/2014/434072

**Published:** 2014-08-10

**Authors:** Xiuchan Guo, Cheryl A. Winkler, Ji Li, Li Guan, Minzhong Tang, Jian Liao, Hong Deng, Guy de Thé, Yi Zeng, Stephen J. O'Brien

**Affiliations:** ^1^Key Laboratory of Laboratory Medicine, School of Laboratory Medicine and Life Science, Wenzhou Medical University, Wenzhou 325000, China; ^2^State Key Laboratory for Infectious Diseases Prevention and Control, Institute for Viral Disease Control and Prevention, Chinese CDC, Beijing 10052, China; ^3^ICF International, Atlanta, GA 30329, USA; ^4^Basic Research Laboratory, Frederick National Laboratory, Leidos Biomedical Research, Inc., National Cancer Institute, Frederick, MD 21702, USA; ^5^Theodosius Dobzhansky Center for Genome Bioinformatics, St. Petersburg State University, St. Petersburg 199004, Russia; ^6^College of Life Science and Bio-Engineering, Beijing University of Technology, Beijing 100022, China; ^7^Cancer Center, Wuzhou Red Cross Hospital, Guangxi 543002, China; ^8^Cangwu Institute for Nasopharyngeal Carcinoma Control and Prevention, Wuzhou, Guangxi 543100, China; ^9^Institut Pasteur, 75724 Paris, France

## Abstract

Genetic factors, as well as environmental factors, play a role in development of nasopharyngeal carcinoma (NPC). A number of single nucleotide polymorphisms (SNPs) have been reported to be associated with NPC. To confirm these genetic associations with NPC, two independent case-control studies from Southern China comprising 1166 NPC cases and 2340 controls were conducted. Seven SNPs in *ITGA9* at 3p21.3 and 9 SNPs within the 6p21.3 *HLA* region were genotyped. To explore the potential clinical application of these genetic markers in NPC, we further evaluate the predictive/diagnostic role of significant SNPs by calculating the area under the curve (AUC). *Results.* The reported associations between *ITGA9* variants and NPC were not replicated. Multiple loci of *GABBR1*, *HLA-F*, *HLA-A*, and *HCG9* were statistically significant in both cohorts (*P*_combined_ range from 5.96 × 10^−17^ to 0.02). We show for the first time that these factors influence NPC development independent of environmental risk factors. This study also indicated that the SNP alone cannot serve as a predictive/diagnostic marker for NPC. Integrating the most significant SNP with IgA antibodies status to EBV, which is presently used as screening/diagnostic marker for NPC in Chinese populations, did not improve the AUC estimate for diagnosis of NPC.

## 1. Background

Nasopharyngeal carcinoma (NPC) is rare in most regions of the world; however, it is a common cancer in Southern China, especially in the Guangdong and Guangxi Provinces. The incidence rate of NPC for males in the Southern Chinese provinces of Guangdong and Guangxi is more than 20 per 100,000 person-years and up to 25–40 per 100,000 person-years in some areas bordering the Xijiang River and Pearl River drainages in these two provinces [1, 2]. It has been well established that Epstein-Barr virus (EBV) is strongly associated with NPC [3–5]. The association of EBV antibodies and NPC were first reported in 1966 [6]. Later, the presence of IgA antibodies to EBV in serum was found to serve as a predictive marker for NPC in Chinese populations [7–9]. IgA antibody titers to the EBV viral capsid antigen (EBV/IgA/VCA) and to the EBV early antigen (EBV/IgA/EA) have been used for the screening and diagnosis of NPC for many years in Southern Chinese populations [7, 9–11]. Epidemiological studies have pointed to other environmental factors (including consumption of salt-preserved fish, exposure to domestic wood-cooking fires, and exposure to occupational solvents) as having a role in development of NPC [5, 12].

Evidence for genetic modulation of NPC risk has accumulated recently. Familial aggregation of NPC cases has been observed in both high- and low-risk populations in different geographic regions [5, 13–15]. Several studies have shown associations between* HLA* genes and NPC [16–21]. The results from our phase I cohort confirm and extend previously reported* HLA* and NPC associations in Southern Chinese populations [22]. Two genome-wide association studies (GWAS) have identified multiple gene association with risk of NPC in Chinese ancestry cohorts [23, 24]. The first GWAS comprised 111 unrelated NPC cases and 260 controls and a replication sample set of 168 cases and 252 controls from the Malaysian Chinese population [23] reported evidence of association with* ITGA9* on Chr 3p21.31-21.2. The second GWAS was conducted in 277 Taiwanese NPC cases and 285 controls and included two independent replication sets. This group found associations with variants on Chr 6p21.3 in or near* HCG9*,* HLA-A*,* HLA-F*, and* GABBR1 *genes [24].

To investigate whether genetic variants can improve the EBV IgA antibodies test method for NPC diagnosis, we extended previously reported GWAS associations with NPC to Han Chinese from Southern China—the highest NPC incidence region. Here, two independent case-control studies were conducted—phase I cohort with 350 NPC cases and 619 controls and phase II cohort with 816 NPC cases and 1721 controls to determine if the polymorphisms of* ITGA9*,* HLA-A*,* HLA-F*,* GABBR1*, and* HCG9* were associated with NPC development or can be potential genetic markers for onset of NPC in a Southern Chinese population.

## 2. Materials and Methods

### 2.1. Cases and Controls (Table 1)

The NPC cohorts were recruited from areas along the Xijiang River in Guangdong and Guangxi Provinces of Southern China in two collection phases [5, 25]. Phase I participants were recruited from April 2000 to June 2001. NPC cases were either incident or prevalent biopsy-confirmed NPC cases. The controls were the case's spouse or geographically matched residents who were NPC-free at the time of study enrollment. Phase II study participants were recruited from November 2004 to October 2005. Cases were incident or prevalent, biopsy-confirmed NPC. Controls were NPC-free at the time of study enrollment and matched to NPC cases on age and district/township of residence. NPC cases were patients at Wuzhou Red Cross Hospital in Wuzhou City and outpatients at Cangwu Institute for NPC Control and Prevention in Cangwu County. All participants self-identified as Han Chinese and reported at least three generations of residency in Guangdong or Guangxi Province, China.

IgA antibodies to EBV capsid antigen (EBV/IgA/VCA) and IgA antibodies to EBV early antigen (EBV/IgA/EA) were determined by serological testing at the time of study enrollment. The cutoff titer for the seropositive status was at least 1 : 10 and 1 : 5 for IgA/VCA and IgA/EA, respectively, based on local standard. Blood samples were obtained from 350 NPC cases (66.6% male) and 619 controls (42.8% male) for phase I; the mean age was 45 years ± 11 and 46 years ± 10 for NPC cases and controls, respectively. For phase II, blood samples were collected from 816 NPC cases (73.2% male) and 1721 controls (61.4% male); the mean age was 45 years ± 11 and 46 years ± 12 for NPC cases and controls, respectively. Family history of NPC, parental ancestry for three generations, dietary and smoking habits, household exposures to wood fires, and occupational exposures to solvents were also captured by questionnaire in the phase II cohort [5]. Participants were asked if there was a family history of NPC in first- (children, siblings, or parents), second- (aunts or uncles, nieces or nephews, and grandparents), or third-degree relatives (first cousins). Information was also collected on the frequency of consumption per month (≥3 times/month, and <3 times/month) of salty fish and preserved meat. Questions on cigarette smoking included current and past smoking habits and number of cigarettes smoked per day. Questions on household and occupational exposures captured data on domestic exposure to wood fires for cooking and occupational exposures to solvents (e.g., formaldehyde, acetone, toluene, or xylene) and duration of exposure (>10 years or ≤10 years). Responses were recorded by double-entry and verification of all data was performed to avoid data entry errors. We excluded persons of minority ethnicity and those who had blood relatives in either the case or control group. We also did not allow overlap in participation between phase I and phase II; the cohorts were independent. Institutional review board approval was obtained from all participating institutions and informed consent was obtained from each study participant.

### 2.2. Genomic DNA Extraction

In phase I participants, DNA was extracted from whole blood or lymphoblastoid cell lines using QIAamp DNA blood maxi kit (Qiagen, Valencia, CA, catalog number 51194). More than 80% of the genotypes were determined from DNA directly extracted from whole blood. In phase II participants, DNA was extracted from whole blood by traditional phenol/chloroform method with Phase Lock Gel tube (Qiagen, MaXtract High Density, catalog number 129065).

### 2.3. Genotyping

In both phases I and II, 7 SNPs of* ITGA9* on 3p21.3 and 9 SNPs within the* GABBR1*,* HLA-F*,* HLA-A*, and* HCG9* genes on chromosome 6p21.3 were genotyped by using commercially available TaqMan SNP genotyping assays and GeneAmp PCR System 9700 (Applied Biosystems, Foster City, CA, USA), in accordance with the manufacturer's instructions. The sequence detection software was used for allelic discrimination. For quality control, 8 to 16 template-free controls, one family sample [25], and 5% to 10% of duplicate samples were included in each 384-well plate.

### 2.4. Statistical Analysis

Hardy-Weinberg equilibrium (HWE) assumptions were independently tested for each SNP in cases and controls for each phase group as well as the two phases combined as a quality control measure. For allele association (Table 2, Supplementary Tables 1 and 2; Supplementary Material available online at http://dx.doi.org/10.1155/2014/434072), the Armitage's trend test was used to calculate the *P* value for additive allele effects on the disease penetrance. ORs were calculated by Mantel-Haenszel estimate based on contingency tables of allele-by-trait counts. For controlling the confounding covariates (age, sex, etc.), the stratified case-control test was performed. All results shown were adjusted for age and sex. In order to exclude the influence of EBV, we analyzed the associations between polymorphisms and the occurrence of NPC using EBV/IgA/VCA and EBV/IgA/EA antibody titers as covariates. For phase II, environmental factors including family history with NPC, consumption of salt-preserved fish, exposure to domestic wood-cooking fires, and exposure to occupational solvents were used as covariates. The receiver operator characteristic (ROC) curve was used to assess the diagnostic performance of EBV/IgA/VCA or EBV/IgA/EA alone, SNP alone, and the integration of these risk factors. Statistics were calculated in the statistical package SAS and SAS Genetics version 9.1.3. Linkage disequilibrium (LD) maps, blocks, and haplotypes were generated by Haploview software [26].

## 3. Results

### 3.1. Association Results with SNPs on* HLA* Region at 6p21.3

As shown in Table 1, over 95% of NPC cases (titer 1 : 10 to 1 : 640) and 42%–45% of the controls (titer 1 : 10 to 1 : 160) were positive for EBV/IgA/VCA antibodies; about 60%–72% of NPC cases (titer 1 : 5 to 1 : 640) and 2%-3% of the controls (titer 1 : 5 to 1 : 80) were positive for EBV/IgA/EA antibodies in the two cohorts. EBV/IgA/EA positive serostatus was always concordant with IgA/VCA seropositive status. To replicate the results of the GWAS showing association between NPC and chromosome 6 [24], 9 SNPs within the* HLA* region previously found to be associated with NPC were genotyped (Table 2). The genotype frequencies for 9 polymorphisms conformed HWE expectations for two control groups; the call rate was 97.9%–99.3% for the 9 SNPs. Table 2 provides the risk alleles, the OR, 95% confidence intervals (CIs), and *P* values for phases I and II combined and phase II controlling for environmental factors. Eight SNPs in* GABBR1*,* HLA-A,* and* HCG9* were significantly associated with NPC in the phase I cohort (Supplementary Table 1: *P* = 0.0001–0.02), phase II cohort (Supplementary Table 1: *P* = 3.09 × 10^−12^–1.13 × 10^−6^), and combined phases I and II (Table 2: *P* = 5.96 × 10^−17^–1.85 × 10^−8^). The SNP on* HLA-F* was significant in phase II and in the combined cohort but not in the smaller phase I cohort, although the ORs were in the same direction. After additionally controlling for EBV/IgA/VCA and EBV/IgA/EA antibody titers, 9 SNPs were also significant (Table 2). For the phase II cohort, after adjusting for sex, age, and EBV antibodies titers, we further controlled for environmental factors, which were shown to be associated with NPC in this cohort [5], including family history with NPC, consumption of salt-preserved fish, exposure to domestic wood-cooking fires, and exposure to occupational solvents as covariates. The 9 SNPs remained significantly associated with the risk of NPC (Table 2, last two columns).

**Figure 1:**
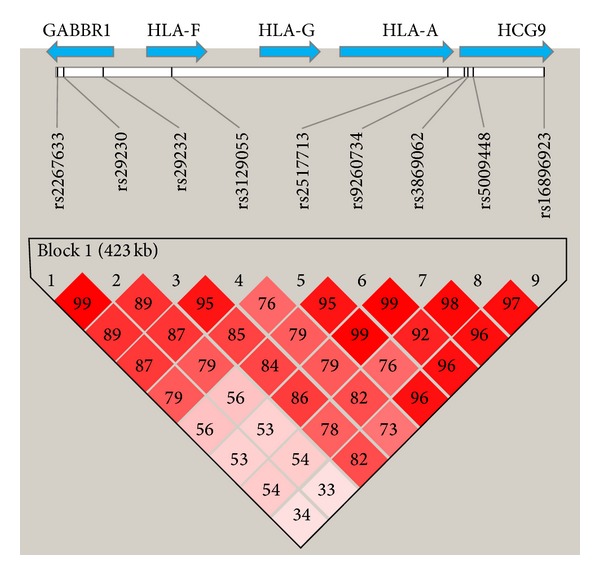
LD map based on *D*′ was drawn using the genotype of the cases and controls.

**Figure 2:**
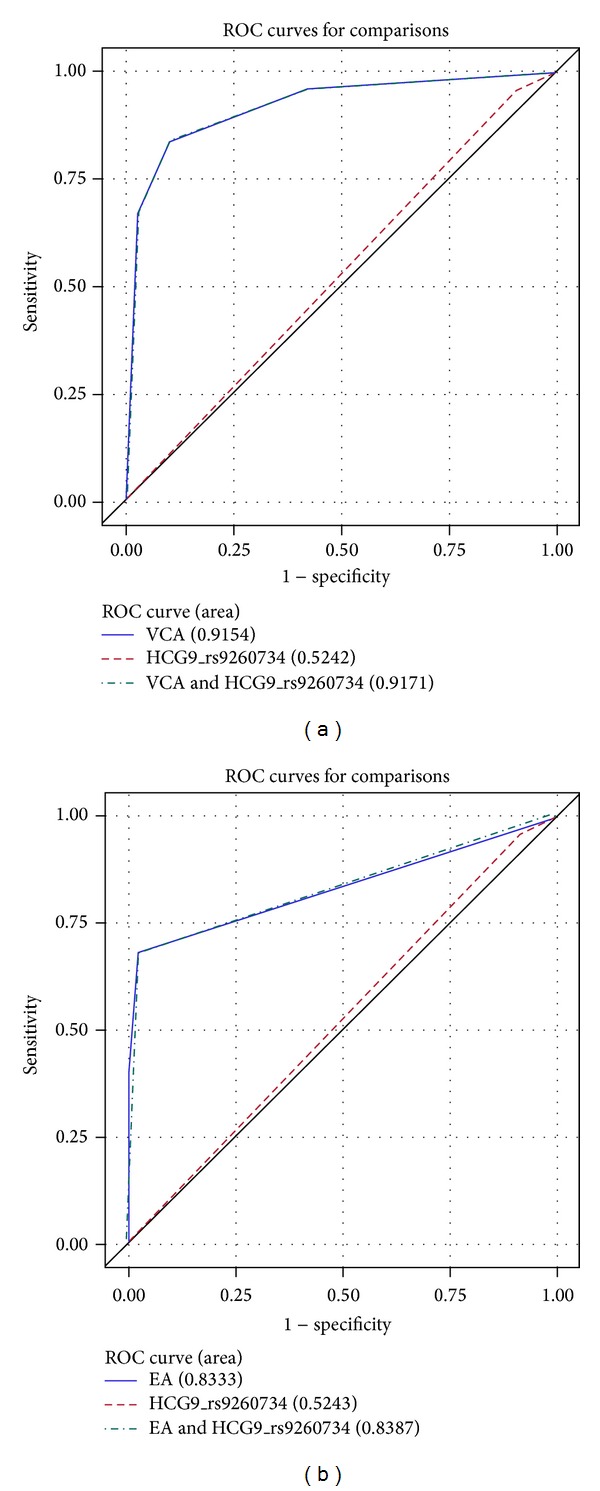
ROC curves.

Based on the LD map all 9 SNPs are in the same block (Figure 1). The* HCG9*-rs9260734, the most significant SNP, was used for evaluating whether the genetic signature can serve as a diagnostic marker for NPC.  Table 3 presented the sensitivity, specificity, and accuracy for EBV/IgA/VCA, EBV/IgA/EA, and SNP test. The specificity (9.3%) and accuracy (38%) of* HCG9*-rs9260734 are lower than 50%, which indicated that the SNP does not qualify as a diagnostic marker. To evaluate if the SNP can improve current IgA antibodies test for NPC diagnosis, we compared the receiver operating characteristic (ROC) curves between IgA/VCA or IgA/EA alone and integrated the SNP with status of IgA/VCA or IgA/EA. The area under the curve (AUC) for integrated markers did improve compared with IgA/VCA or IgA/EA alone (0.917 versus 0.915 for IgA/VCA; 0.833 versus 0.839 for IgA/EA; Figures 2(a) and 2(b)); however, this was not statistically significantly different.

### 3.2. Association Results with SNPs on* ITGA9* at 3p21.3

To examine the influence of* ITGA9* gene variants on NPC [23], 7 SNPs in* ITGA9* were genotyped in phase I and phase II cohorts. Each of the 7 SNPs confirmed to HWE expectations in controls for both cohorts and the genotype call rate was 96.8%–99.4%. No evidence of association was seen between 6 of the SNPs and NPC in phase I or phase II or in the combined analysis (Supplementary Tables 1 and 2). SNP (rs169111) was modestly significant in the combined analysis (Supplementary Table  2: OR = 1.4, *P* = 0.03) but not after adjusting for EBV IgA antibodies titers.

## 4. Discussion

In our study, we have demonstrated the strong associations of 9 SNPs located within* GABBR1*,* HLA-F*,* HLA-A*, and* HCG9* with NPC. Our results, from two independent Han Chinese NPC cohorts, confirm the previous associations and effect sizes reported in the Taiwanese GWAS [24]. The etiology of nasopharyngeal carcinoma is influenced by both genetic and environmental factors. EBV IgA antibody status is a strong predictive marker and plays an important role in NPC development in Southern Chinese populations [9, 10, 27, 28]. Our results show that over 95% of NPC cases were EBV/IgA/VCA antibody positive and about 60%–72% of NPC cases were positive for EBV/IgA/EA antibodies. To exclude potential influence of EBV antibody status, we controlled EBV/IgA/VCA and EBV/IgA/EA antibody titers during the analysis. The results indicated that the variants of* GABBR1*,* HLA-F*,* HLA-A*, and* HCG9* were still associated with NPC. In a previous study we reported that family history with NPC, consumption of salt-preserved fish, exposure to domestic wood-cooking fires, and exposure to occupational solvents were risk factors of NPC [5]. These environmental exposure data were available in our phase II cohort. When we adjusted for these factors in our association analysis, the 95% confidence intervals overlapped indicating that variants on* GABBR1*,* HLA-F*,* HLA-A*, and* HCG9* association were independent of environmental factors with NPC onset; the reduced statistical significance in the analysis adjusted for environmental factors reflects the small sample size with environmental data. These results, from two independent cohorts, affirm the associations of* GABBR1*,* HLA-F*,* HLA-A*, and* HCG9* with NPC in the Han Chinese population.* HLA-A* and* HCG9 *have been reported to be associated with EBV-positive Hodgkin lymphoma and infectious mononucleosis caused by EBV infection [29, 30]. This suggested that genetic variation chromosome 6p21.3 can influence the outcome of primary EBV infection and the level of viral persistence. A genome-wide expression profiling has revealed that increased EBV gene expression is strongly associated with inhibition of multiple* HLA* class I gene expression in NPC [31], further implicating these genes with NPC.

EBV/IgA/VCA and EBV/IgA/EA antibody titers, especially EBV/IgA/VCA, have been used for the screening and diagnosis of NPC for over 30 years in Southern Chinese populations [7, 9–11]. Along with the cancer genetic/genomic project development, new genetic variants associated with NPC are continuously being discovered. There is a growing need to evaluate the genetic markers for medical practice. Our interest is whether the significant SNPs can serve as a diagnostic marker or improve the IgA antibodies test for NPC prediction. Sensitivity, specificity, and accuracy are widely used statistics to quantify how good and reliable a test is. The receiver operating characteristic (ROC) curve is a graphic presentation of the relationship between both sensitivity and specificity and becomes the standard analytical tool for evaluating diagnostic tests. In this study, the specificity and accuracy of SNP was lower than 50%; the AUC of SNP was less than 0.6 indicating SNP alone cannot be a diagnostic marker for NPC. Considering the positive rate for EBV/IgA/VCA antibodies in the general population is about 3% [10], we randomly selected samples and made the control group that contained 3% IgA/VCA positive, then we repeated the ROC analysis; we obtained similar results. The AUC increased when IgA/VCA or IgA/EA was integrated with SNP; however, the effect was not statistically significant. To our knowledge, this is the first study to explore and incorporate the genetic variants to clinical use for NPC. Our results show that there is a strong association between variants of* GABBR1*,* HLA-F*,* HLA-A*, and* HCG9* and NPC, but they cannot be useful for individualized risk prediction/diagnosis of NPC. However, risk profile based on a combination of genetic and other risk factors leads to an appreciable increased risk of disease and there is potential for increased predictive power as more genetic risk variants are detected [32].

We were unable to confirm the reported association with NPC of 7 SNPs in* ITGA9* in our large Chinese Han population (*N* = 3506) from an NPC high incidence region of Southern China, although this study was well powered to detect a similar level of association. Based on our results, the minor allele frequencies (MAFs) for these 7 SNPs were lower (between 0.024 and 0.03). The previous study was carried out on a relatively small sample with 279 cases and 512 controls [23]; the few patients and a low MAF may have contributed to low statistical power for SNP association. However, two more recent NPC GWAS also failed to replicate the association of* ITGA9* with NPC [33, 34].

Our study is unique in that two critical EBV antibody titers, as well as environmental factors, are available for inclusion in the statistical modeling. We controlled for these risk factors to determine the genetic association with NPC. This study systematically replicates the association from two NPC GWAS [23, 24] performed in NPC high-risk Asian populations. We also explored potential clinical use for significant genetic makers. Further study should focus on how these genetic variants impact function at the molecular and cellular levels to affect NPC development. Understanding the functional consequences of genetic variation will be critical to advancing our knowledge of the etiology of the disease and implementing rational medical strategies.

## 5. Conclusion

In summary, our results extend the association of the* GABBR1*,* HLA-F*,* HLA-A*, and* HCG9* locus with NPC to the Han Chinese population of Southern China. We show for the first time that these factors are independent of environmental risk factors previously shown to influence NPC development. This study also indicated that EBV IgA antibodies in combination with these genetic makers may not be useful for diagnostic prognosis of NPC.

## Supplementary Material

Supplemental Data comprises two tables. Table 1 provides the association between SNPs at 3p21.3, 6p21.3 and NPC in phase I and phase II cohorts, respectively including the OR, 95% confidence intervals (CIs) and P values. Table 2 provides the association between SNPs in the ITGA gene at 3p21.3 and NPC in phase I and phase II combined cohort and includes the OR, 95% confidence intervals (CIs) and P values.

## Figures and Tables

**Table 1:** Characteristics of participants in a study of nasopharyngeal carcinoma (NPC) in southern China.

	Phase I		Phase II	
	Cases	Controls	Cases	Controls
Age (years)	45 ± 11.4 (SD)	46 ± 9.7 (SD)	45 ± 11.0 (SD)	46 ± 11.7 (SD)
Male, %	66.6 (233/350)	42.8 (265/619)	73.2 (597/816)	61.4 (1056/1721)
IgA/VCA+*, %	95.4 (334/350)	44.7 (277/619)	95.8 (782/816)	42.3 (731/1721)
IgA/EA+∗∗, %	59.5 (201/348)	2.3 (14/619)	72.4 (591/816)	2.6 (44/1721)

Total	350	619	816	1721

Age: the age at diagnosis of NPC for cases and age of enrollment for controls.

SD: standard deviation.

∗Positive for IgA antibodies to Epstein-Barr virus capsid antigen.

∗∗Positive for IgA antibodies to Epstein-Barr virus early antigen.

The cutoff value for seropositive status is 1 : 10 for IgA/VCA and 1 : 5 for IgA/EA.

**Table 2:** Association between alleles of SNPs at 6p21.3 and NPC in phase I and phase II combined.

Gene-SNP	Risk allele	Phase I and phase II		Phase I and phase II		Phase II	
		OR (95% CI)∗	P*	OR (95% CI)∗∗	P**	OR (95% CI)∗∗∗	P***
GABBR1-rs2267633	A	1.61 (1.41–1.84)	1.02*E* − 12	1.48 (1.17–1.87)	0.001	1.41 (1.02–1.95)	0.03
GABBR1-rs29230	T	1.64 (1.45–1.89)	1.36*E* − 13	1.61 (1.28–2.04)	6.14*E* − 05	1.61 (1.16–2.22)	0.004
GABBR1-rs29232	A	1.35 (1.21–1.49)	1.85*E* − 08	1.41 (1.16–1.71)	0.0006	1.33 (1.01–1.76)	0.05
HLA-F-rs3129055	G	1.14 (1.02–1.28)	0.02	1.33 (1.09–1.64)	0.008	1.47 (1.10–1.20)	0.01
HLA-A-rs2517713	T	1.61 (1.43–1.82)	2.44*E* − 16	1.69 (1.35–2.08)	2.58*E* − 06	1.64 (1.20–2.22)	0.003
HCG9-rs9260734	G	1.67 (1.47–1.87)	5.96*E* − 17	1.75 (1.41–2.17)	6.48*E* − 07	1.75 (1.28–2.44)	0.0005
HCG9-rs3869062	A	1.60 (1.42–1.81)	3.4*E* − 14	1.63 (1.30–2.04)	1.97*E* − 05	1.60 (1.16–2.19)	0.004
HCG9-rs5009448	C	1.62 (1.45–1.82)	1.89*E* − 16	1.66 (1.33–2.06)	3.46*E* − 06	1.64 (1.20–2.26)	0.002
HCG9-rs16896923	T	1.54 (1.35–1.75)	4.56*E* − 11	1.69 (1.33–2.13)	2.19*E* − 05	1.64 (1.18–2.27)	0.005

OR: odds ratio. CI: confidence interval.

∗Adjusted for sex and age.

∗∗Additionally adjusted for EBV/IgA/VCA and EBV/IgA/EA titers.

∗∗∗Additionally adjusted for EBV/IgA/VCA and EBV/IgA/EA antibody titers and other environmental factors including family history with NPC, consumption of salt-preserved fish, exposure to domestic wood cooking fires, and exposure to occupational solvents.

**Table 3:** The diagnosis performance of IgA/VCA and IgA/EA and genetic signature.

Test	Sensitivity (%)	Specificity (%)	Accuracy (%)
EBV/IgA/VCA	95.7	56.9	69.8
EBV/IgA/EA	68.6	97.5	87.9
HCG9-rs9260734	95.6	9.3	38.0

Note: the threshold for IgA/VCA is 1 : 10 and for IgA/EA is 1 : 5.
