# Supplementary material for: Evaluation and Integration of Genetic Signature for Prediction Risk of Nasopharyngeal Carcinoma in Southern China
Source: Biomed Res Int. 2014 Aug 10;2014:434072. doi: 10.1155/2014/434072 (PMC4142549; doi:10.1155/2014/434072)
Supplement: Supplementary file 1 — Supplemental Data comprises two tables. Table 1 provides the association between SNPs at 3p21.3, 6p21.3 and NPC in phase I and phase II cohorts, respectively including the OR, 95% confidence intervals (CIs) and P values. Table 2 provides the association between SNPs in the ITGA gene at 3p21.3 and NPC in phase I and phase II combined cohort and includes the OR, 95% confidence intervals (CIs) and P values. [file 434072.f1.pdf]

Supplemental Data

Supplementary table 1: Genetic association between alleles of SNPs at 3p21.3, 6p21.3 and NPC in phase I and phase II cohort

	Phase I		Phase II	
Gene-SNP	OR (95%CI)	*P*	OR (95%CI)	*P*
ITGA9-rs169188	0.65 (0.36-1.17)	0.14	0.74 (0.50-1.09)	0.13
ITGA9-rs197721	1.39 (0.80-2.40)	0.22	1.34 (0.93-1.92)	0.12
ITGA9-rs149816	1.45 (0.82-2.58)	0.18	1.43 (0.98-2.11)	0.07
ITGA9-rs169111	1.42 (0.81-2.47)	0.20	1.52 (1.02-2.25)	0.04
ITGA9-rs197770	0.64 (0.35-1.15)	0.12	0.73 (0.50-1.06)	0.10
ITGA9-rs189897	1.38 (0.79-2.40)	0.24	1.26 (0.86-1.84)	0.23
ITGA9-rs197757	1.55 (0.84-2.84)	0.15	1.29 (0.87-1.91)	0.20
GABBR1-rs2267633	1.59 (1.22-2.07)	0.0005	1.60 (1.37-1.88)	3.53E-09
GABBR1-rs29230	1.56 (1.19-2.0)	0.0009	1.67 (1.43-1.96)	1.73E-10
GABBR1-rs29232	1.36 (1.10-1.67)	0.004	1.36 (1.20-1.54)	1.13E-06
HLA-F-rs3129055	1.05 (0.84-1.19)	0.64	1.19 (1.04-1.37)	1.01E-02
HLA-A-rs2517713	1.52 (1.20-1.89)	0.0002	1.63 (1.43-1.87)	5.87E-12
HCG9-rs9260734	1.54 (1.22-1.96)	0.0003	1.67 (1.45-1.92)	3.09E-12
HCG9-rs3869062	1.43 (1.12-1.82)	0.003	1.62 (1.40-1.87)	1.71E-10
HCG9-rs5009448	1.56 (1.24-1.97)	0.0001	1.58 (1.37-1.81)	1.11E-10
HCG9-rs16896923	1.33 (1.03-1.72)	0.02	1.54 (1.33-1.82)	3.76E-08

OR: odds ratio. CI: confidence interval.

Odds ratios (OR) were calculated by Mantel-Haenszel test based on contingency tables from cases versus controls, adjusted for sex and age.

P value of Armitage's trend test from cases versus controls, adjusted for sex and age.

Supplementary table 2: Genetic association between alleles of SNPs in ITGA9 at 3p21.3 and NPC in phase I and phase II combined

	Phase I and Phase II		Phase I and Phase II	
SNP ID	OR (95%CI)*	*P**	OR (95%CI)**	*P***
rs169188	0.75 (0.54-1.03)	0.07	1.02 (0.58-1.79)	0.95
rs197721	1.28 (0.95-1.72)	0.10	0.90 (0.54-1.51)	0.69
rs149816	1.34 (0.98-1.84)	0.06	0.85 (0.50-1.44)	0.54
rs169111	1.40 (1.02-1.91)	0.03	0.85 (0.51-1.42)	0.53
rs197770	0.74 (0.54-1.01)	0.06	1.13 (0.68-1.89)	0.62
rs189897	1.24 (0.91-1.69)	0.17	1.04 (0.61-1.77)	0.88
rs197757	1.29 (0.94-1.79)	0.12	1.11 (0.64-1.92)	0.69

OR: odds ratio. CI: confidence interval.

*Adjusted for sex and age

** Additionally adjusted for EBV/ IgA/VCA and EBV/ IgA/EA titers
